# The Teeth

**Published:** 1890-12

**Authors:** 


### THE TEETH.

*Good Housekeeping* treats of various subjects relating to the family. In a late number it has this to say of the teeth: The temporary teeth should have the best possible care. Their function is an important one ; they are to " hold the fort " till the permanent set are ready to come upon the scene, and should then give way to their successors with the cheerfulness of a displaced politician. It is, therefore, a mistake to suppose that on account of their temporary character their decay is a matter of little consequence, or that they may be extracted at any time without injury. They should be kept in the best condition possible until the development of their successors absorb their roots, and they become loosened. If this loosening fails to take place, as frequently occurs, they should be drawn as soon as the crown of the permanent tooth appears through the gum, in order that the latter may take its proper place in line. Two or three times a year is not too often to have a dentist examine the mouth of a child, till the permanent teeth have developed.

The first molar of each set—known as the six-year molar—may appear any where from five to seven years of age, and this, besides being the first of the permanent teeth, is also specially liable to decay. Very generally it is the first tooth requiring the dentist's forceps, and may be drawn before the 12-year molar of the same set makes its appearance. In this case the loser, as the cavity will be partially filled by other teeth when they appear, often believes that his quota of teeth has been less than his neighbor's. Even parents often confound these molars with the temporary set and neglect them when known to be diseased, supposing they will soon give place to others. The second set of molars appears at about the age of 12, and the last of wisdom teeth, five or six years later. The advent of any of these is liable to be accompanied by soreness, ulceration, or more serious complications ; the eyes or ears may be affected, or serious nervous troubles may result. When these or similar complications arise, not readily understood, it is well to look for the cause in the mouth. One of the most common affections of the teeth is tartar, a deposit which comes from the saliva and the various impurities with which it is mingled, forming an adherent crust which may be almost flint like in hardness, or so soft that it may easily be removed with a finger nail ; the color, also, may vary from black to white, through nearly all gradations. Where, as often happens, the formation insinuates itself between the gums and the teeth, it may work irreparable mischief, and whenever a deposit is noticed, it should have prompt attention at the hands of the dentist—not only for its removal, but for the correction, if possible, of the cause.

It cannot be too strongly impressed that any diseased condition of the teeth should at once have treatment, and this for a variety of reasons, not all of which receive the attention that is their due. It is not only necessary in order to prevent more extended ravages, but the general health must unavoidably suffer. The breath of a person with diseased teeth is often so offensive as to sicken those who chance to inhale it, and, of course, correspondingly mortifying to the sufferer. But this is not all, nor the worst. The offensive particles, which render expirations so offensive, must inevitably be carried to the lungs, where their effect will as inevitably be felt, sooner or later ; and, mingled with the food and drink, they go to the stomach, whence their rank poison, absorbed in the circulation, permeates the system with deadly influence. How important, then, that cleanliness and constant care of the mouth and teeth should be exercised as a preventive of more serious disorders of the general system.

